# Peripapillary hyperreflective ovoid mass-like structures with cystoid macular edema: a case report

**DOI:** 10.1186/s12886-024-03509-3

**Published:** 2024-06-11

**Authors:** Wei Liu, Jianjun Yan, Hao Huang

**Affiliations:** 1 Department of Ophthalmology, Zhuzhou Hospital Affiliated to Xiangya School of Medicine, Central South University, 116 South Changjiang Road, Zhuzhou, 412000 China; 2 State Key Laboratory of Ophthalmology, Zhongshan Ophthalmic Center, Guangdong Provincial Key Laboratory of Ophthalmology and Visual Science, Guangdong Provincial Clinical Research Center for Ocular Diseases, Sun Yat-Sen University, 54 South Xianlie Road, Guangzhou, 510060 China

**Keywords:** Peripapillary hyperreflective ovoid mass-like structures (PHOMS), Cystoid macular edema (CME), Case report

## Abstract

**Background:**

Peripapillary hyperreflective ovoid mass-like structures (PHOMS) are newly characterized lesions wedged around the optic discs, which used to be misdiagnosed. Better understanding and identifying PHOMS are important for monitoring the condition of optic nerve.

**Case presentation:**

A young female presented to the ophthalmic clinic with blurred vision of both eyes. Protrusions resembling “C-shaped donut” were found circling the optic discs bilaterally. These lesions were homogenous hyperreflective on OCT, while they were also hypoautofluorescent and hypoechogenic. Meanwhile, cystoid macular edema (CME) was also identified in both eyes. The patient was then diagnosed as PHOMS with CME. A short-term glucocorticoids therapy was prescribed systemically. The logMAR best-corrected visual acuity (BCVA) of both eyes reached 0.0 in 4 months with recovery of CME, while the PHOMS remained.

**Conclusions:**

There is currently no report on PHOMS with CME. More attentions should be paid to PHOMS, for they are potential biomarkers for axoplasmic stasis involved in different diseases of the optic nerve.

**Supplementary Information:**

The online version contains supplementary material available at 10.1186/s12886-024-03509-3.

## Introduction

Peripapillary hyperreflective ovoid mass-like structures (PHOMS) are a set of newly defined lesions recognized to be a sign of axoplasmic stasis in the optic papilla [1]. Used to be labelled as a group of optic disc drusen (ODD) subtypes, PHOMS were identified independent in 2018 [1, 2].

The pathological changes of PHOMS are considered to be abnormal manifestations of hernias resulted from the distended axons of retinal ganglion cells [3], and they usually present themselves in “donut” or “torus” shapes. Distinct from ODD, PHOMS are protrusions embedded between the nerve fiber layer (NFL) and Bruch’s membrane peripapillary circling the optic discs on optical coherence tomography (OCT), with homogenous hyperreflectivity [4]. The hypoechogenicity and hyperautofluorescence provides PHOMS with more characteristics [5].

PHOMS might be accompanied by the onset of different diseases involving the optic nerve and retina, where axoplasmic flow is commonly compromised [5]. However, the pathological mechanism of PHOMS is not clarified. Accordingly, recognition of PHOMS is essential, not only for avoiding misdiagnosis as ODD, but also for better observation on the pathophysiological processes of the optic nerve.

Up to the present day, few reports about PHOMS have been published, while researching study is even rare. Here, we report a case of bilateral PHOMS with cystoid macular edema (CME) for the first time.

### Case presentation

**Fig. 1:**
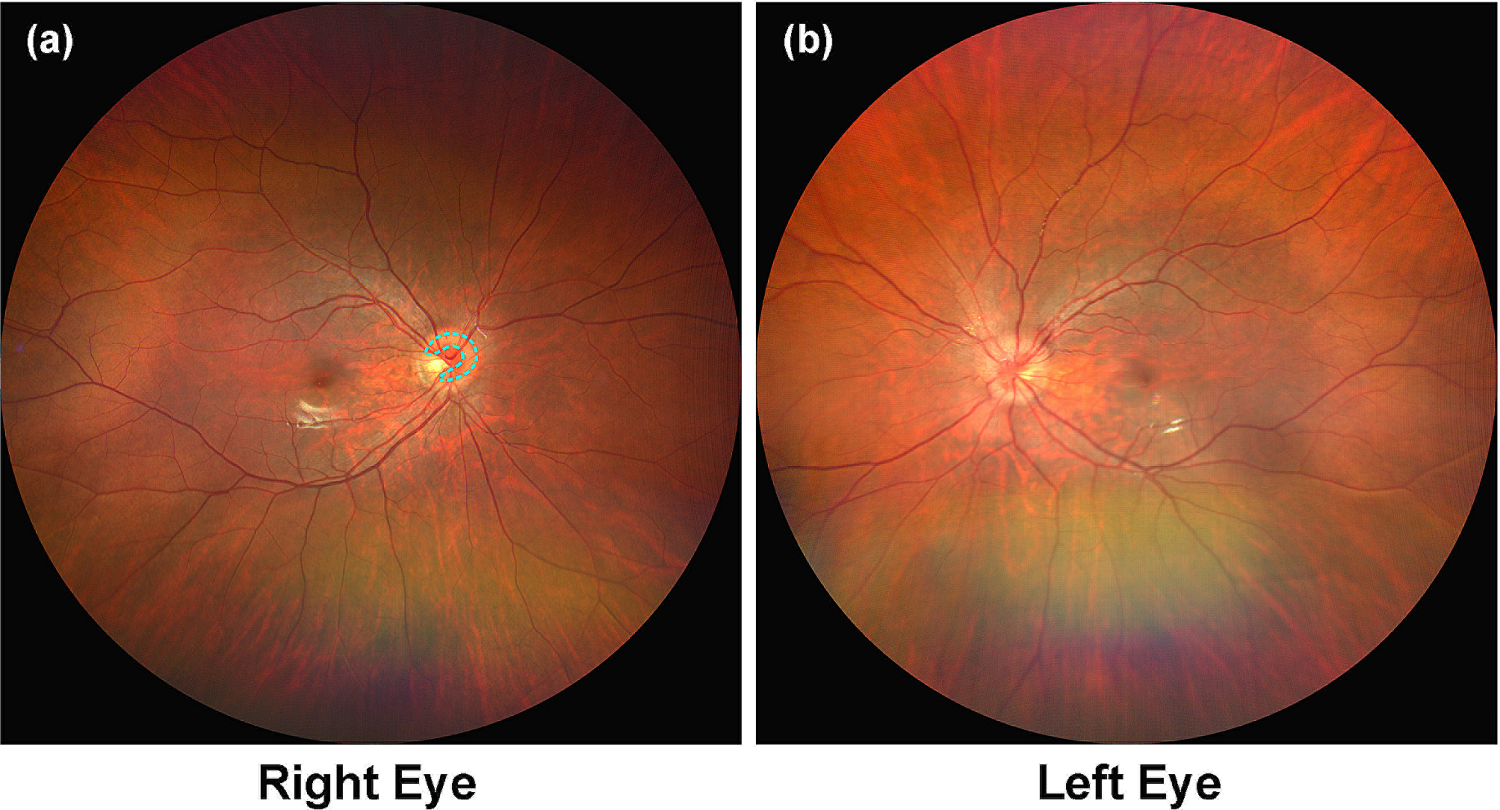
Fundus photography showing the retinal images. (**a**-**b**) The patient’s optic papillae were symmetrically protuberant in both eyes. PHOMS resembling“C-shaped donuts” lay under the retina and circled the optic nerve heads, resulting in blurred disc margins (blue dashed line)

An 18-year-old female presented to the ophthalmic clinic with bilateral blurred vision in a short term. The decline of vision was mild but in a gradual progress. The patient denied past medical history but myopia. No systemic anomaly was found. The logMAR best-corrected visual acuity (BCVA) of the right eye was 0.1 (-2.0 diopters), and that of the left was 0.4 (-3.0 diopters). The intra-ocular pressure (IOP) maintained in a normal range. With slit-lamp examination, there was no cell found in her anterior chamber, nor abnormality occurred in the anterior segments. A slight amount of cells in the vitreous cavity were observed with funduscope. Both of her optic papillae were protuberant with unclear margins, with a construction resembling the “C-shaped donut” hid under each of the discs (Fig. 1). Though the other parts of her retinas were normal, the foveal reflection was also reduced in both eyes.

**Fig. 2:**
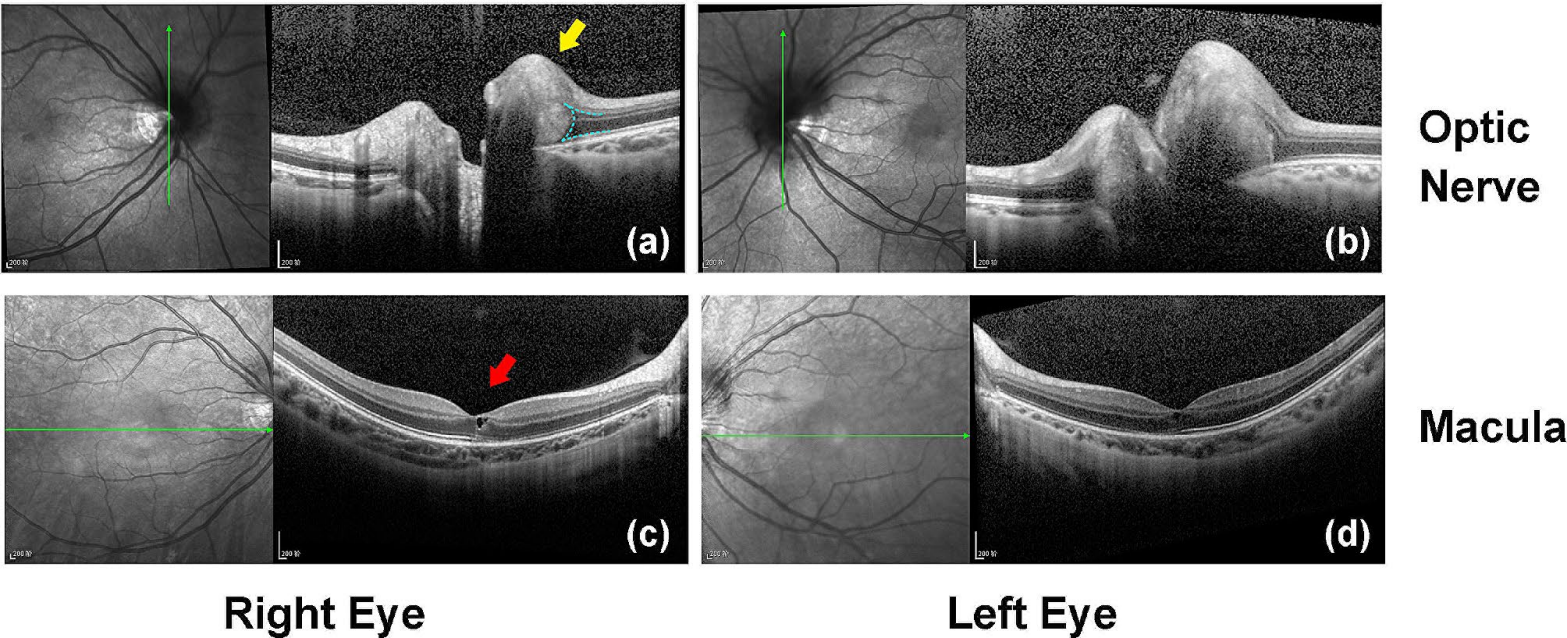
EDI-OCT showing the morphologies of optic papillae and maculae. (**a**-**b**) PHOMS were peripapilary, bulging between the RNFL and Bruch’s membrane (yellow arrow). The signal inside PHOMS were diffusively hyperreflective. The inner retinal layers were elevated by the PHOMS, forming a “ski slope-shaped” reflective region of the RNFL (blue dashed line). (**c**-**d**) Bilateral CME were found at the first visit (red arrow), accompanying enhanced reflection of the posterior choroids

**Fig. 3:**
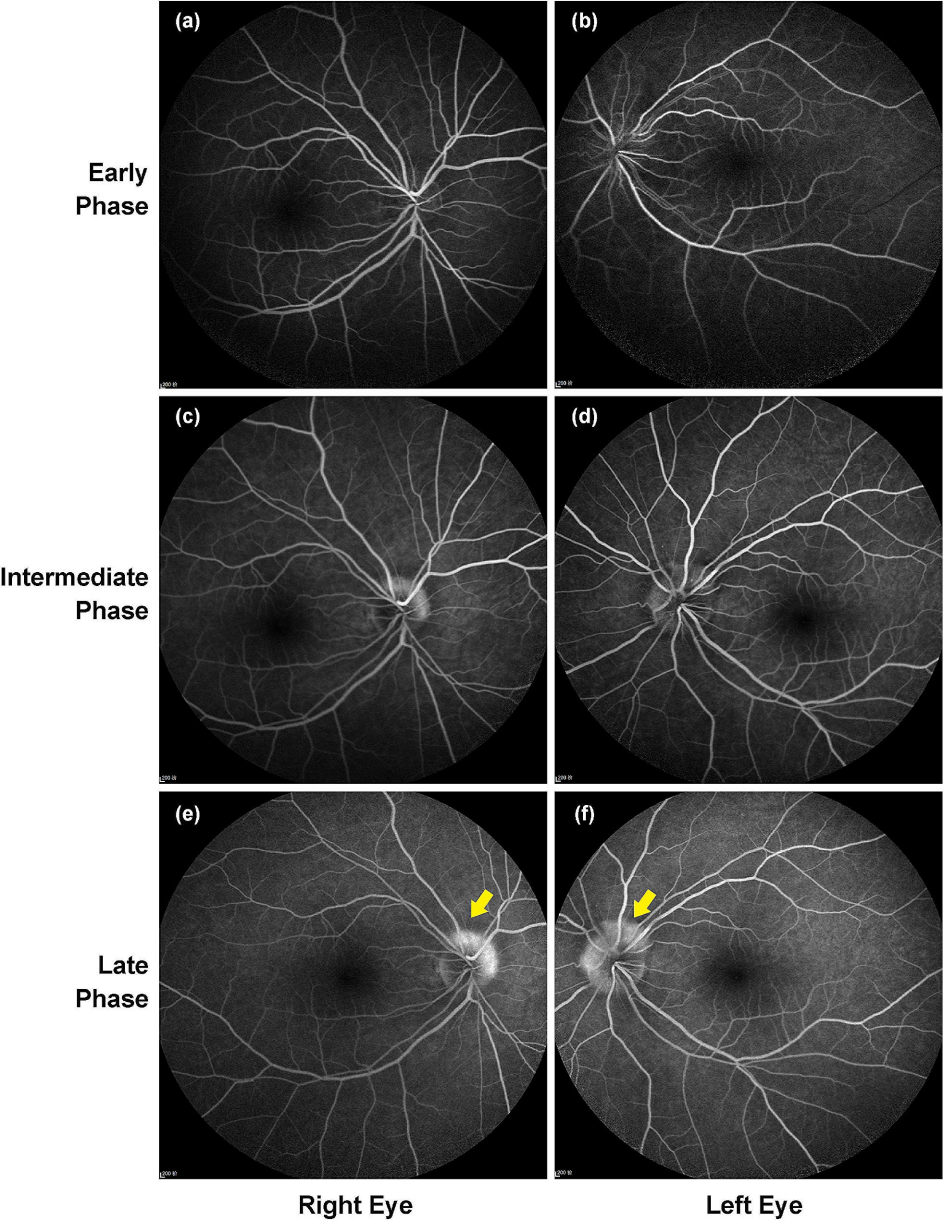
Fundus fluorescent angiograph (FFA) at the first visit. (**a**-**b**) Retinal arteriovenous circulation time was normal. The PHOMS was hypofluorescent at the early phase. (**c**-**f**) Over time, staining of the PHOMS emerged and enhanced (yellow arrow). No fluorescein leakage was seen

Further inspections were carried out. The protrusions were internally hyperreflective in homogeneity on enhanced depth imaging (EDI) - OCT, forming crateriforms around the asymmetric optic nerve heads (Fig. 2). The lesions of ovoid structure were wedged between the RNFL and Bruch’s membrane, lifting the inner retinal layers. Bilaterally, CME and enhanced reflectivity of the posterior choroid were confirmed as well (Fig. 2). Staining of the lesions were observed in the late stage of fluorescein angiography, while there were no anomalous signs found in the retinal circulation time and vascular fluorescence (Fig. 3). The lesions were hypoautofluorescent in fundus imaging and hypoechogenic in B-mode ultrasound. No obvious visual field defect or abnormal visual evoked potential (VEP) were found. Systemic examinations showed low signals of the lesions in the bone window sections of computed tomography (CT) (Figure S1). On magnetic resonance imaging (MRI), normal images were observed in her central nervous system (CNS), while the optic papillary lesions remained indiscernible (Figure S2). The patient refused examinations on cerebrospinal fluid or intracranial pressure, while she reported no symptoms of CNS. Therefore, craniocerebral diseases were not consider. Further investigations excluded infectious diseases, nervous diseases, autoimmune diseases, neoplastic diseases, and other systemic diseases. It was firstly confusing for the lesions resembled to the expression of ODD in fundus examinations. Nevertheless, the lesions were identified as PHOMS according to the criteria (Table 1) [4, 5]. Accompanied bilateral CME was also diagnosed. In this case, compressive effects on the optic papilla caused by PHOMS was considered to be a possible cause of CME, but there was no direct evidence at the first visit. Though no definite predisposing factor was confirmed for the edema, the hyperreflectivity in the posterior choroid indicated inflammation. Therefore, short-term systemic glucocorticoids were prescribed: initiating with 240 mg/d and 120 mg/d of intravenous methylprednisolone (each dosage lasted for 3 days), the patient received tapering oral prednisone from 80 mg/d, 40 mg/d, 20 mg/d, 10 mg/d to 5 mg/d for 1 month in the subsequent treatment.

**Table 1:** Criteria to define PHOMS [4, 5]

Properties	Examination	Presentation
Location	OCT	Peripapillary
		Above Bruch’s membrane
	B-scan	Gap aligned with the optic disc as the center
Appearance	OCT	Signal deflection resembling a “ski slope” crossing more than one retinal layer
		Signal strength similar to retinal nerve fiber and ganglion cell layers
		Homogenous hyperreflectivity internally

Four months later, the logMAR BCVA of her both eyes raised to 0.0. EDI-OCT showed restoration of the macula, while the PHOMS and RNFL thickness remained unchanged (Figure S3). This outcome also excluded the possibility of CME caused by PHOMS compression, for the remaining of PHOMS should have continuous impact on the optic construction in inducing CME. Moreover, the vitreous cells were dissipated. Neither evidence of worsening conditions nor systemic discomforts were reported.

## Discussion and conclusions

PHOMS were distinguished as a sign of axoplasmic stasis by the *ODD Consortium* in 2018 [1]. Though they used to be mistakenly diagnosed, the uniform hyperreflectivity of PHOMS on OCT distinguishes these lesions from the combining hyperreflective shell and hyporeflective core of ODD. While PHOMS are non-calcified, the morphology is usually in a shape of bulging loop circling the optic disc [4]. The affected discs exhibit blurring boundaries that might be misdiagnosis as neuropapillitis or papilloedema in funduscope, so that OCT examination is required for diagnosis.

In a report from the Copenhagen Child Cohort 2000 Eye Study, a prevalence of 8.9% was found in healthy children [6]. Another case report described the thinning RNFL in 3 pediatric patients with PHOMS [7]. Notwithstanding, the etiology of PHOMS is not yet clear and no targeted therapy is available. It is hypothesized to be associated with ODD, papilloedema, anterior ischaemic optic neuropathy, inflammatory demyelinating disorders, retinal vein occlusion, diabetic papillopathy, optic nerve compression, enlarging cavernous venous malformation, acute Leber’s hereditary optic neuropathy, or other unclear ocular diseases [5, 8–11]. PHOMS were also reported to be linked to myopia, tilted optic nerve head, and prelaminar hyperreflective lines on OCT [6]. Therefore, the most likely inference for the PHOMS reported in the present case is the tilted disc syndrome resulted from myopia. Meanwhile, the choroidal inflammation should also be taken into a possible inducement. Given that PHOMS are potential signs of axoplasmic stasis and the potentially relevant diseases [3], more attentions should be paid to further explore its characteristics and prognosis.

A former case described PHOMS in a 68-year-old male with nonarteritic anterior ischaemic optic neuropathy dissolved in 5 months [5]. Another study reported that most of PHOMS disappeared with resolved axoplasmic stasis [8]. In eyes with acute Leber’s hereditary optic neuropathy, only a small number of cases had residual PHOMS after 12 months [10]. Interestingly, the PHOMS maintained unchanged after therapy in the present case. This could be a result of short tracking time, or irreversible tilted optic nerve head caused by myopia. In this case, continuous observation is of value. The blurred vision, however, was strongly linked to the CME, for her visual acuity improved obviously along with macular recovery. In the initial judgment, we could not rule out the possibility that her CME was caused by the compression of PHOMS on optic papilla. Nevertheless, the CME was relieved by the glucocorticoid therapy while the PHOMS remained. This result indicated that it was not the compressive effect but posterior inflammation had given rise to the CME.

General to the best of our knowledge, PHOMS with CME is unusual in clinical practices. By reporting this case, we hope to provide an example of the disease database and raise the awareness of the optic neuropathy prone to misdiagnosis.

### Electronic supplementary material

Below is the link to the electronic supplementary material.

Supplementary Material 1

Supplementary Material 2

Supplementary Material 3

Supplementary Material 4

## Data Availability

No datasets were generated or analysed during the current study.

## Abbreviations

BCVA: Best-corrected visual acuity
CME: Cystoid macular edema
CNS: Central nervous system
CT: Computed tomography
EDI: Enhanced depth imaging
IOP: Intra-ocular pressure
MRI: Magnetic resonance imaging
OCT: Optical coherence tomography
ODD: Optic disc drusen
PHOMS: Peripapillary hyperreflective ovoid mass-like structures
RNFL: Retinal nerve fibre layer
VEP: Visual evoked potential
