# Supplementary material for: Peripapillary hyperreflective ovoid mass-like structures with cystoid macular edema: a case report
Source: BMC Ophthalmol. 2024 Jun 11;24:247. doi: 10.1186/s12886-024-03509-3 (PMC11165800; doi:10.1186/s12886-024-03509-3)
Supplement: Supplementary file 1 — Supplementary Material 1 [file 12886_2024_3509_MOESM1_ESM.docx]

**Supplemental figure legends**

**Figure S1.** CT imaging showing that the PHOMS were hyporeflective, in consistent with the other soft tissues.

**Figure S2.** MRI imaging showing that the PHOMS were indiscernible both in T1 and T2 weighted images.

**Figure S3.** Follow up on the optic papillae and maculae with EDI-OCT. (a-b) Treatment of glucocorticoids didn’t alter the morphology of the PHOMS on EDI-OCT. (c-d) The CME significantly ameliorated after the treatment, and the choroidal reflection was also decreased.
