# Supplementary figures and images for: Peripapillary hyperreflective ovoid mass-like structures with cystoid macular edema: a case report
Source: BMC Ophthalmol. 2024 Jun 11;24:247. doi: 10.1186/s12886-024-03509-3 (PMC11165800; doi:10.1186/s12886-024-03509-3)

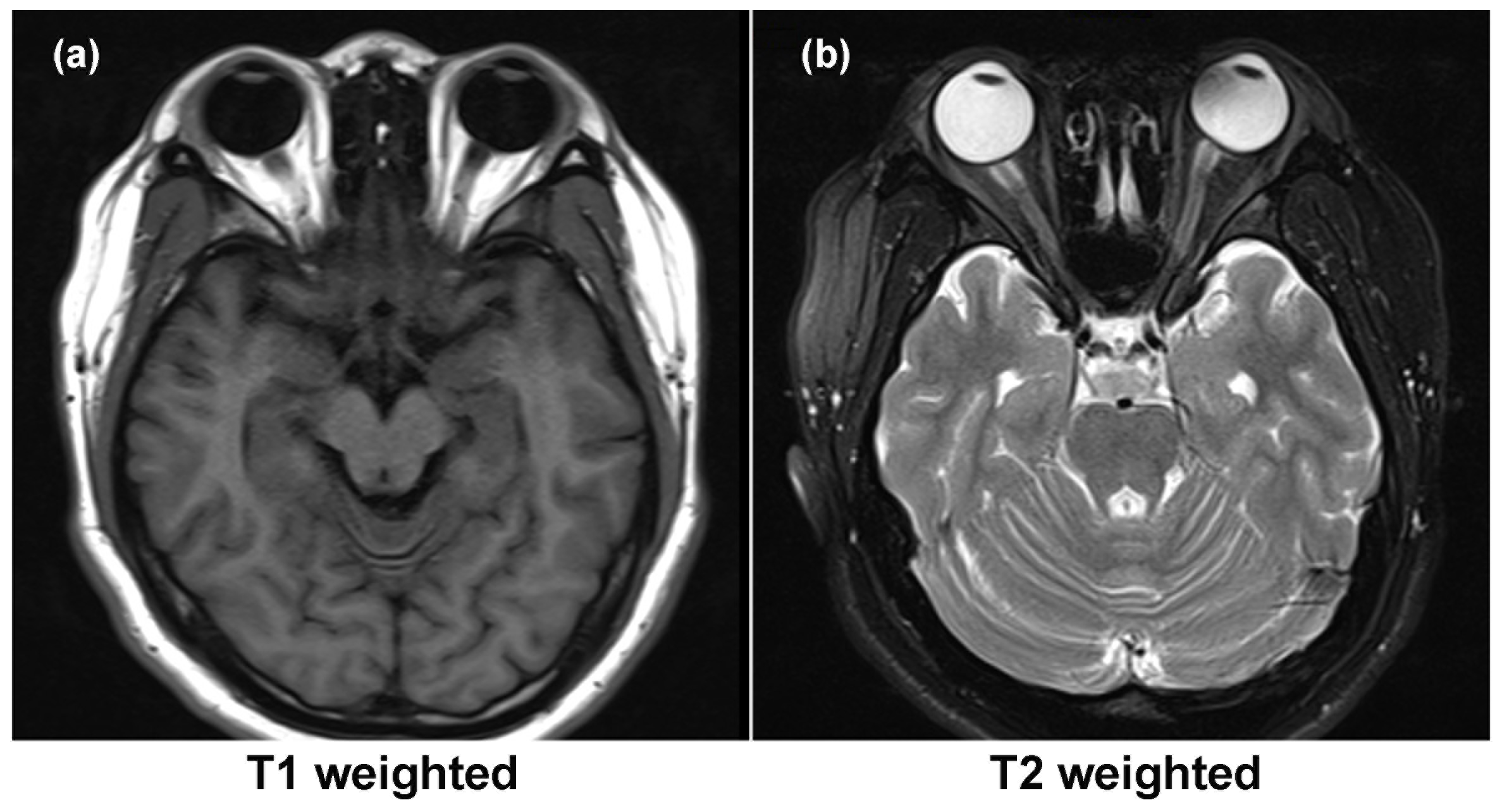

Supplement: Supplementary file 2 — Supplementary Material 2 [file 12886_2024_3509_MOESM2_ESM.png]

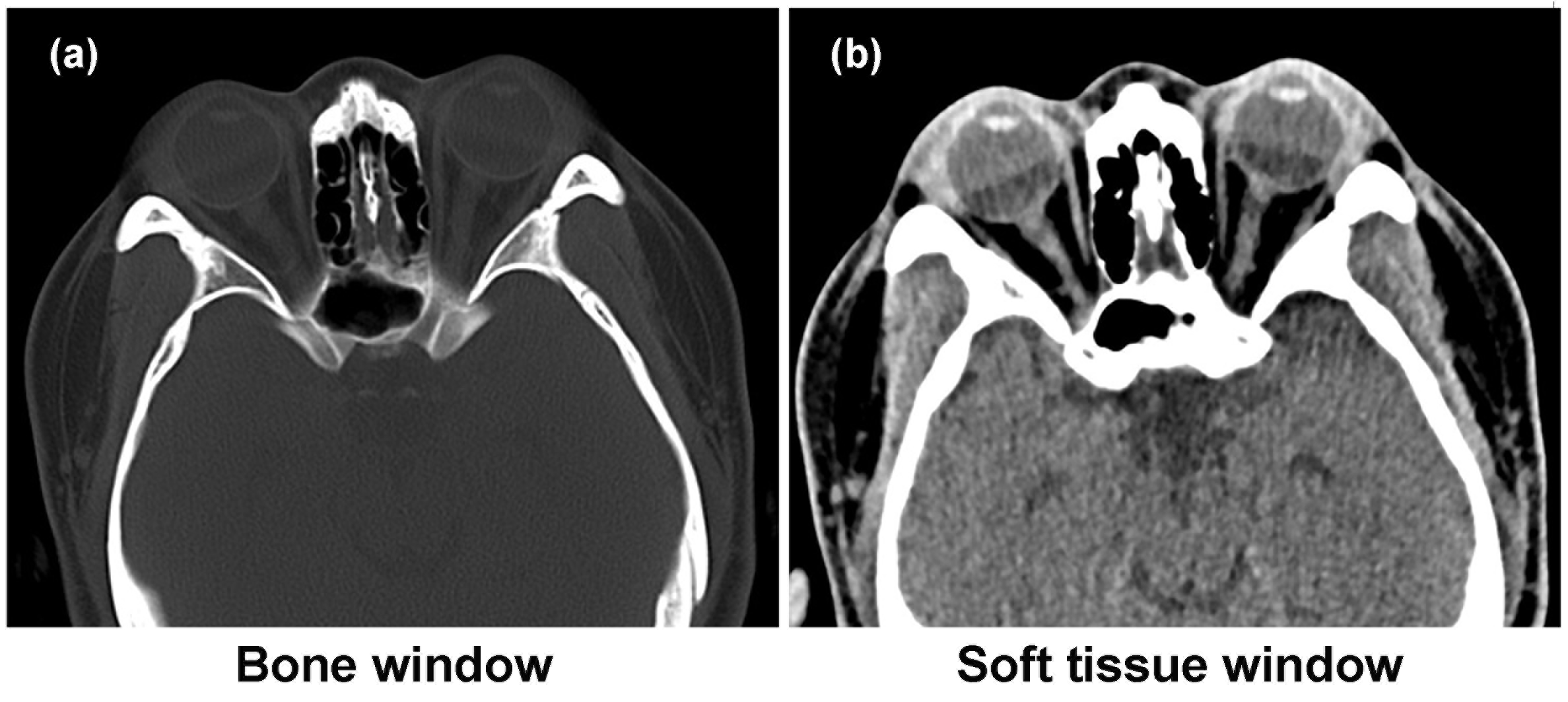

Supplement: Supplementary file 3 — Supplementary Material 3 [file 12886_2024_3509_MOESM3_ESM.png]

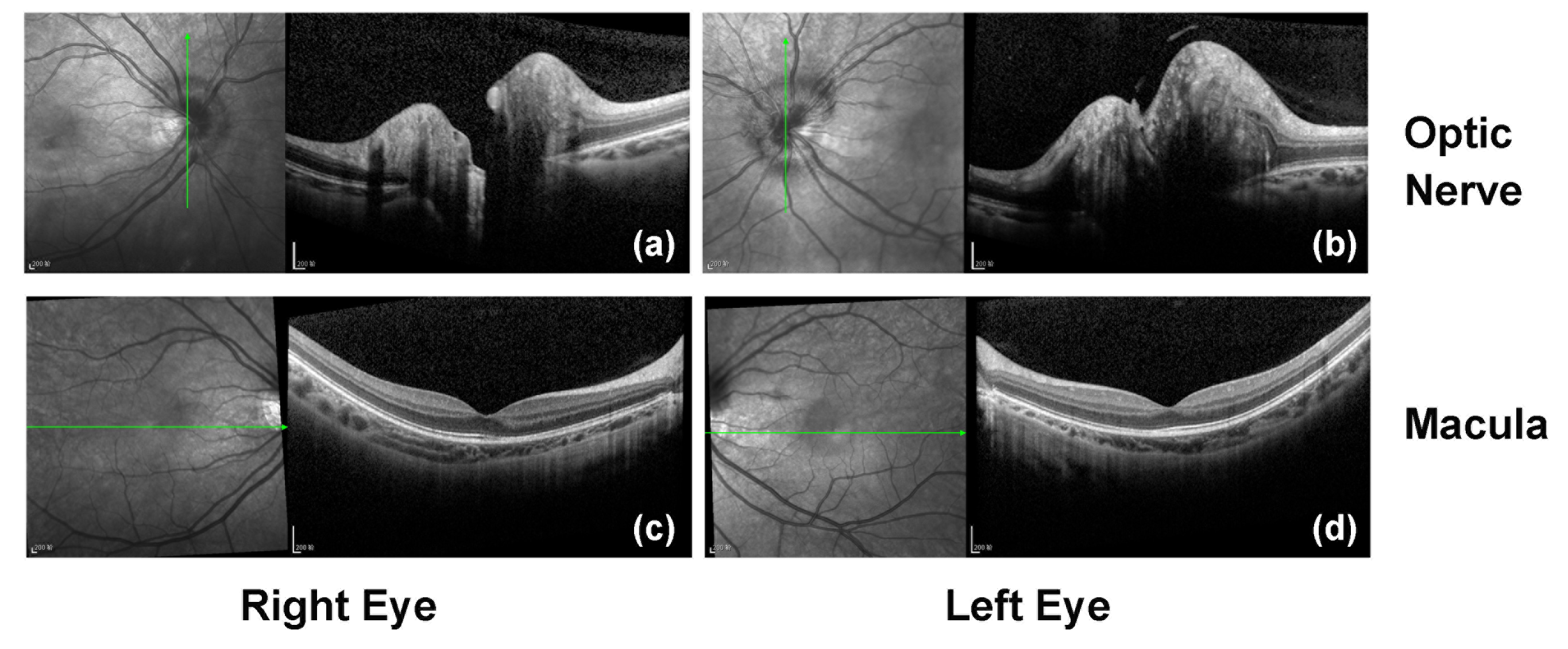

Supplement: Supplementary file 4 — Supplementary Material 4 [file 12886_2024_3509_MOESM4_ESM.png]
